# 

**Published:** 1886-07

**Authors:** 


### OUR ADVERTISERS.

FELLOWS' SYRUP HYPOPHOSPHITES.—This preparation is being extensively prescribed by the profession everywhere, and so far as we are aware, with entire satisfaction. It has sustained a high reputation for efficiency in the treatment of pulmonary affections, and is employed also in various nervous and debilitating diseases with success.

MASSANETTA WATER.—Elsewhere will be found the advertisement of this popular mineral water. Dr. D. Caldwell Ireland, of Baltimore, thus writes of the water: "I very cheerfully recommend the use of Massanetta water as a curative agent in the treatment of malarial fevers. I have used it with decided benefit in my own case, and have employed it with patients for the past three years with good results."

SEABURY & JOHNSON, NEW YORK.—These gentlemen present in this issue an attractive advertisement, which will be found on third cover page. Their goods are unequalled by any manufacturer we know. See the advertisement and write them for any samples desired.

BRADFIELD & WARE, ATLANTA.—This firm is one of the best in the State. Besides keeping a first-class prescription store, they have all the best mineral waters on draught. Their soda water is unexcelled by any in the city.

E. FUGERA & CO., NEW YORK.—This firm stands deservedly high. Among their many excellent preparations they have a pure pepsin, put up in small soluble capsules, which can be preserved indefinitely. This pepsin contains neither starch nor sugar of milk. It will digest ninety times its weight of meat. Write them for samples and try it. You will be pleased.

THE PIEDMONT AIR-LINE—RICHMOND AND DANVILLE RAILROAD SYSTEM.—This famous line of through-travel to the East has already become the most popular route, not only to Washington, Baltimore, Philadelphia, New York and all Eastern cities, owing to increased facilities in the way of solid trains to Washington city, fast schedules, elegant day coaches, close connections, etc., but is doing an immense business to all summer resorts of North Georgia, Virginia and the Carolinas. The grand and picturesque scenery of the French Broad Valley and the Western North Carolina mountains are on the Western North Carolina Division of the Richmond and Danville Road. The various resorts located in this lovely mountain section are gaining greater popularity each season, and are destined to extend their fame over the entire country. New hotels are being built at various places, others are being enlarged, and every effort made to accommodate the hundreds of guests who flock there to enjoy the cool breezes and life giving waters of this Switzerland of America. The Piedmont Air-line gives choice of two different routes to Western North Carolina resorts. Since the completion of the Asheville and Spartanburg Railroad, the shortest possible route has been opened to the public from the South. The increased schedule and additional line of sleepers via Salisbury makes the trip a pleasant one to the tourist whose leisure does not demand the shorter distance and quicker transit. Tallulah Falls, the rival of sublime Niagara, is still a point of great attraction. Increased facilities for summer travel have been made, and the rush will now be greater than ever before. All the Virginia springs are reached by this line. The new management have placed the PIEDMONT AIR-LINE in a position far superior to all its competitors, and the appreciative public will accord to it that increase of freight and passenger business it so justly deserves.

